# An automated data cleaning method for Electronic Health Records by incorporating clinical knowledge

**DOI:** 10.1186/s12911-021-01630-7

**Published:** 2021-09-17

**Authors:** Xi Shi, Charlotte Prins, Gijs Van Pottelbergh, Pavlos Mamouris, Bert Vaes, Bart De Moor

**Affiliations:** 1 Department of Electrical Engineering (ESAT), Stadius Centre for Dynamical Systems, Signal Processing and Data Analytics, KU Leuven, Kasteelpark Arenberg 10 - Box 2446, 3001 Leuven, Belgium; 2 Leuven Statistics Research Center, KU Leuven, 3000 Leuven, Belgium; 3 Academic Center for General Practice, KU Leuven, 3000 Leuven, Belgium

**Keywords:** Data cleaning, Automated method, Clinical decision support

## Abstract

**Background:**

The use of Electronic Health Records (EHR) data in clinical research is incredibly increasing, but the abundancy of data resources raises the challenge of data cleaning. It can save time if the data cleaning can be done automatically. In addition, the automated data cleaning tools for data in other domains often process all variables uniformly, meaning that they cannot serve well for clinical data, as there is variable-specific information that needs to be considered. This paper proposes an automated data cleaning method for EHR data with clinical knowledge taken into consideration.

**Methods:**

We used EHR data collected from primary care in Flanders, Belgium during 1994–2015. We constructed a Clinical Knowledge Database to store all the variable-specific information that is necessary for data cleaning. We applied Fuzzy search to automatically detect and replace the wrongly spelled units, and performed the unit conversion following the variable-specific conversion formula. Then the numeric values were corrected and outliers were detected considering the clinical knowledge. In total, 52 clinical variables were cleaned, and the percentage of missing values (completeness) and percentage of values within the normal range (correctness) before and after the cleaning process were compared.

**Results:**

All variables were 100% complete before data cleaning. 42 variables had a drop of less than 1% in the percentage of missing values and 9 variables declined by 1–10%. Only 1 variable experienced large decline in completeness (13.36%). All variables had more than 50% values within the normal range after cleaning, of which 43 variables had a percentage higher than 70%.

**Conclusions:**

We propose a general method for clinical variables, which achieves high automation and is capable to deal with large-scale data. This method largely improved the efficiency to clean the data and removed the technical barriers for non-technical people.

## Background

With the rapid development of electronic systems, the concept of Electronic Health Records (EHR) have already been widely accepted and used, and the use of EHR data in clinical research is incredibly increasing, leading to “a new era of data-based and more precise medical treatment” [1]. EHRs may include a wide range of data, including socio-demographics, medical history, medication, allergies, immunization and laboratory test results. The abundancy of data resources provides sufficient information for clinical studies, but also raises challenge of data cleaning.

The data collected from daily practice may include lots of typos and errors. The data cleaning of real-world data is time-consuming, and requires to master techniques of data processing. Therefore, a tool for non-technical people, which can do the data cleaning automatically, can save a considerable amount of time and budget.

In order to correctly clean the EHR data and evaluate data quality, some studies gave their own definitions of dimensions of Quality of Data (QoD) for EHR data. A systematic review conducted by Weiskopf et al. in 2013 summarized 5 dimensions, i.e. completeness, correctness, concordance, plausibility, and currency [2]. A study in 2017 summarized accuracy, completeness, consistency, credibility, and timeliness [3], while a study in 2019 assessed primary care EHR data considering comparability, completeness, correctness, and currency [4]. The systematic review in 2018 summarized 34 studies with a focus on data quality assessment in emergency medical services [5], classifying the assessment indicators in all these studies into 5 dimensions, namely completeness, accuracy, consistency, accessibility, and timeliness. The sequence of these dimensions is based on the frequency of occurrence in 34 studies. With the clearly defined dimensions of QoD, some researchers proposed the frameworks to assess the data quality systematically. Kahn et al. [6] presented a “fit-for-use” conceptual model for data quality assessment, and generated a framework to assess the data and control the data quality. The following studies developed their own frameworks to systematically assess data, by calculating self-defined assessment indicators [7, 8], visualizing variability [9], or adding analysis-specific data quality evaluation requirements [10]. However, these frameworks were only meant to assess the data quality, no data cleaning procedures were conducted.

With the necessity of efficiently cleaning EHR data, several proposals for a general framework were made. A study in 2016 applied the framework proposed by Kahn et al. by defining assessment indicators following their 5 domains and correcting the data if there is room to improve the assessment indicators [11]. Weiskopf et al. continued their study in 2013 and published a data quality assessment guideline in 2017 [12], which can help evaluate the data quality from different aspects and hence help solve data issues. The framework presented in the work of Miao et al. functions in a similar way [13]. These methods or frameworks rely on the data quality assessment to accurately identify the potential data issues in the original data, then the researchers worked to solve the data issues accordingly. Manual investigation was throughout the whole process.

Phan et al. [14] proposed an automated data cleaning method for EHR data, but it only considered anthropometric data (height and weight measurements), so that the automated cleaning techniques were based on the data characteristics of height and weight measurements. Tang et al. [15] developed a pipeline with high automation that can process different types of variables, such as categorical variables, numeric variables, and hierarchical variables. But the main functions of this pipeline is to help extract data, select high-quality features, harmonize data, and merge the data as the final output. Data cleaning for wrongly input values was not discussed.

An automated data cleaning method for different types of medical variables is still lacking. Besides, the current automated data cleaning tools in other areas often use a statistical calculation to process all variables uniformly. However, in clinical practice, there is a lot of variable-specific information that needs to be considered. Therefore, it is necessary to develop an automated method with good clinical relevance. The goal of this paper is to introduce an automated data cleaning method, which was used to clean data collected from Belgian primary care.

## Methods

### Study setting

Intego is a longitudinal database of EHR data collected from patients in general practice from the Flanders region in Belgium. It is a unique operational computerized registration network. The network was founded in 1994 and 111 general practitioners (GP) participated by 2015. Data is yearly collected for every patient who had a contact with his GP in that year. In total, about 300,000 individual patients have been recorded in the database since the network started, corresponding to a yearly coverage of approximately 2% of the Flemish population [16]. In 2015, there were more than 3 million clinical measurements, 3 million diagnosis, 14 million prescriptions, and 38 million laboratory tests of about 10,000 variables.

Table 1 shows some example rows of the measurement dataset. The dataset including laboratory tests has a very similar structure. Although Intego also has a prescription dataset and diagnosis dataset, there are no unexpected errors, so they will not be discussed in the paper.

**Table 1:** An example of raw data

Patient ID	GP ID	Birth year	Sex	Test code	Date	Year	Test name	Num1	Num2	Unit
27898	GP_9811	1957	F	S00000057	09/08/2014	2014		165	0	cm
27898	GP_9811	1957	F	S00000058	09/08/2014	2014		56.5	0	kg
27898	GP_9811	1957	F	S00000057	29/09/1995	1995	Height	163	0	S002
27898	GP_9811	1957	F	S00000058	29/09/1995	1995	Weight	61	0	S006
42716	GP_9812	1963	M	S00000069	18/11/2013	2013	1	130	88	mmHg
61905	GP_9845	1964	F	S00000069	16/08/1993	1993	Blood pressure	90	50	mmHg

There were over 10,000 laboratory test variables in Intego. It was not possible to clean all variables. The variables used for testing the automated data cleaning method were determined based on feature selection studies that will be conducted in the future. To avoid bias caused by disease selection, we considered variables related with 3 diseases of different types. We selected and cleaned 52 clinical variables with more than 100,000 observations that were discussed in previous studies about Type 2 Diabetes, myocardial infarction, and rheumatoid arthritis.

### Data descriptions and issues

The main data issues for data cleaning are situated in the values and units in the records of measurements and laboratory tests.

#### Values out of normal range

There are two types of value errors. The first type of errors was caused by the fact that more or less zeros than expected were entered. Take blood pressure as an example, the wrong values can be 9, which should be corrected into 90, or 16,000, which should be corrected into 160. This type of errors can be easily converted back to the normal range by a simple transformation in the order of magnitude. The second type of errors is the unreasonable values that cannot be converted back to the normal range using simple transformation. For instance, 3000 for blood pressure is an unreasonable value even after converted as 30 or 300. This kind of errors should be detected as outliers and replaced with NA. Therefore, it is necessary to know the normal range and outlier range for each variable if we would like to clean the data following real clinical practice.

#### Inconsistent units

There are also two reasons leading to inconsistent units. First, the lab tests can come from different labs across the country, and the labs and GPs are not using uniform units for the same variable. For example, the clinical variable Creatinine has units such as mg/dL, mmol/L, mg%, which are all correct units but should be converted into a uniform format. Second, some of the units were wrongly spelled, e.g. ‘g/dL and mmol/mol, or meaningless, e.g. ??, 77, 1, NULL, etc.. In order to have uniform units, the wrongly spelled units should be automatically detected and replaced with correct units, and then all the units of one variable are converted into one uniform unit. One difficulty of unit conversion is that the conversion rates between the same units for different variables are different. For instance, the conversion rate between mmol/L and mg/dL for glucose is 18 while it is 11.312 for creatinine. Thus, the variable-specific information should be known in advance for better data cleaning.

### Clinical knowledge database (CKD)

The Clinical Knowledge Database (CKD) stores all the data related with variable-specific rules that is necessary for data cleaning. In our use case, we included the conversion formula between different units, and the normal range and extreme range of the clinical variables, however, it is not necessary to keep CKD in a uniform format all the time. It can be easily adapted to other studies if more variable-specific information is needed. For example, it can store the variable-specific statistical measures to aggregate the repeated measure.

The unit conversion rates between all possible units for a certain clinical variable were automatically derived from the website http://unitslab.com/ without manual selection. The standard unit, the normal range, and the extreme range of clinical variables were chosen based on literature and clinical knowledge. The normal range is the reference range for the healthy people and the extreme range is the biologically plausible range. The clinical information can be easily filled in by clinicians after this automated cleaning tool is put into use. Table 2 gives an example of CKD and the full CKD is uploaded online (See Availability of data and materials).

**Table 2:** Example rows in clinical knowledge database

Variable code	Variable name	Variable unit	Conversion rate	Standard unit	Normal range		Extreme range	
					Min	Max	Min	Max
57139A.B	Creatinine	Mg/dL	1	Mg/dL	0.6	1.3	0.1	5
		Mmol/L	11.312	Mg/dL				
		Mg/L	0.1	Mg/dL				

### Fuzzy search

In order to correct the typos in the units, the fuzzy matching algorithm based on Levenshtein distance was applied to automatically detect the wrongly spelled units and replace them with correct units or NA if no similar units could be found. Levenshtein distance is the minimum edit distance that contains a set of editing operations, including changing characters, deleting characters and/or inserting characters. When the words are identical, the distance is zero. When one letter has to be changed/deleted/inserted, the distance becomes one. Therefore, three types of errors are defined, i.e., deletion, insertion, and substitution. Denote *V* and *W* as two words containing letters *i* = 1, …, *n* and *j* = 1, …, *m*, and $\epsilon$ϵ is the empty word, the Levenshtein distance between these words for different types of errors can be defined as the equations below [17].

$$\begin{aligned} Lev\left( {V,\epsilon } \right) & = \left| V \right| = n \\ Lev\left( {\epsilon ,W} \right) & = \left| W \right| = m \\ Lev_{V,W} \left( {i,j} \right) & = \left\{ {\begin{array}{*{20}l} {\max \left( {i,j} \right){ }} \hfill & {i = j} \hfill \\ {min\left\{ {\begin{array}{*{20}c} {1.{\text{ Deletion}}:{ }Lev\left( {i - 1,j} \right) + 1} \\ {2.{\text{ Insertion}}:{ }Lev\left( {i,j - 1} \right) + 1} \\ {3.{\text{Substition}}:{ }Lev\left( {i - 1,j - 1} \right) + 1} \\ \end{array} } \right.} \hfill & {i \ne j} \hfill \\ \end{array} } \right. \\ \end{aligned}$$

The Levenshtein distance has the disadvantage when comparing words at different length, since longer words are more likely to have larger distance. A similarity ratio can be calculated from Levenshtein distance to standardize the distance and make it independent of the word length [18]. The obtained ratio ranges between 0 and 1, with 0 meaning no similarities and 1 meaning identical. The equation for the similarity ratio can be written as below, where *Lev* is the Levenshtein distance, *|V|* and *|W|* are the number of characters. Table 3 gives an example of the similarity ratios between some units.

**Table 3:** An example of the similarity ratio for the unit check

Original unit	Correct unit	Score
106/L	X10exp6/L	0.71
mmol/LsOms	mmol/L	0.75
g/24u14g/L15	g/24u	0.61
77	g/24u	0
24	g/24u	0.57

$$Lev.ratio\left(V,W\right)=1-\frac{Lev(V,W)}{\mathrm{max}(\left|V\right|,\left|W\right|)}$$

**Fig. 1:**
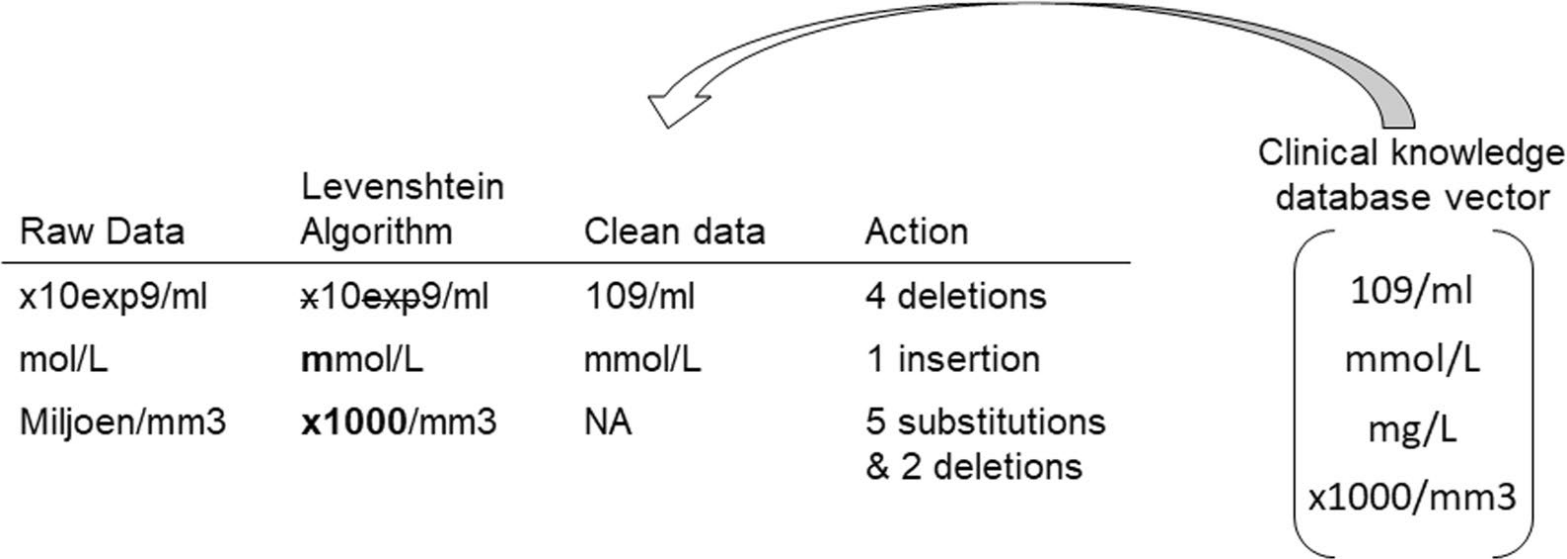
A toy example to show how the fuzzy search method works. The correct possible units for one specific variable are known in CKD. The algorithm compares the raw data and the possible units in CKD and selects one unit that requires a minimum of edits or NA if the scores are below the threshold. (The vector of correct unit is not the full vector in CKD.)

Figure 1 gives a toy example how the fuzzy search works in the real dataset. All possible units for one specific variable are stored in the column *Variable Unit* in CKD. The algorithm computes the scores between the original unit and every possible unit of this variable and replaces the original unit with the correct unit, which has the largest similarity ratio. If the original unit is correctly spelled, there will be one identical unit in CKD, then the original unit will be replaced with this identical unit, i.e., keeps unchanged. Next, for incorrectly spelled units the similarity ratio is set at a specific threshold (in this paper at 0.5). If the similarity ratio is smaller than the threshold, the original unit is replaced with NA.

### Outlier detection

Traditional outlier detection methods that depend on the data distribution to automatically detect values strange or far from the majority of the values could not perform well in our use case. Intego is representative of the general population, so both healthy people and patients are included. If the variables have a large range in values, healthy people and patients can have very different observations. As a consequence, traditional outlier detection methods will detect patients’ records as outliers if the disease prevalence is low, which is almost always the case in primary care.

Van den Broeck et al. [19] suggested an outlier detection method based on variable-specific information. They specified the true normal, true extreme, erroneous and idiopathic values for each variable and discussed the outliers case by case. Their method could not easily be applied in a big data and automated framework, thus, it was simplified into an outlier detection method based on the normal range and extreme range of variables.

**Fig. 2:**
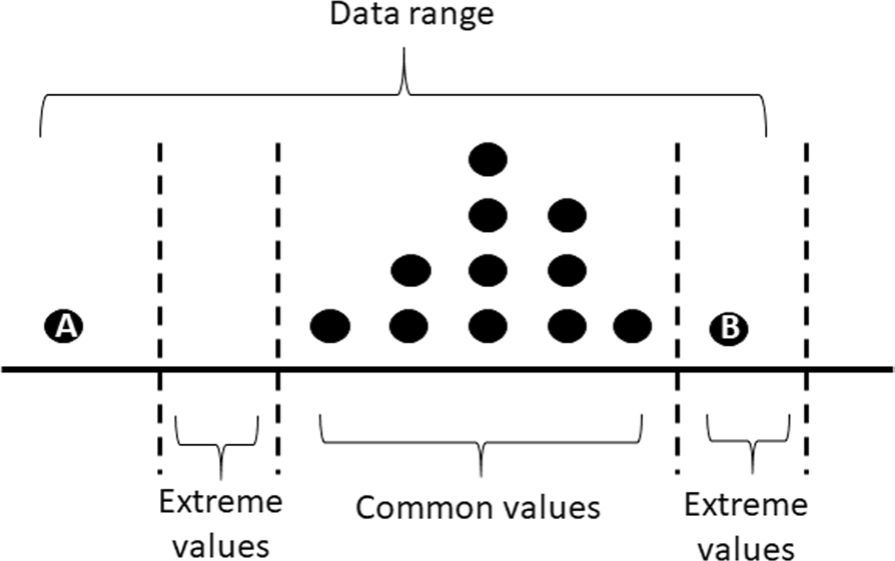
Example of a data range. It is assumed that the majority of the data falls within the common range. All data outside the extreme range are outliers. This makes data point A an outlier and data point B a more extreme but acceptable observation

If the values were out of the normal range, a transformation in the order of magnitude was first conducted, trying to make the values as close to the normal range as possible, e.g. 9 for blood pressure could be converted into 90 (Type 1 error in Section *Data Descriptions and Issues*). If the values were still out of the extreme range after the transformation, they were regarded as outliers and replaced with NA (Type 2 error in Section *Data Descriptions and Issues*), e.g. 3000 for blood pressure was replaced with NA because it is an unreasonable value even when it was converted as 30 or 300. Figure 2 shows an example of the outlier detection by using the data range of clinical variables.

### The whole cleaning framework

**Fig. 3:**
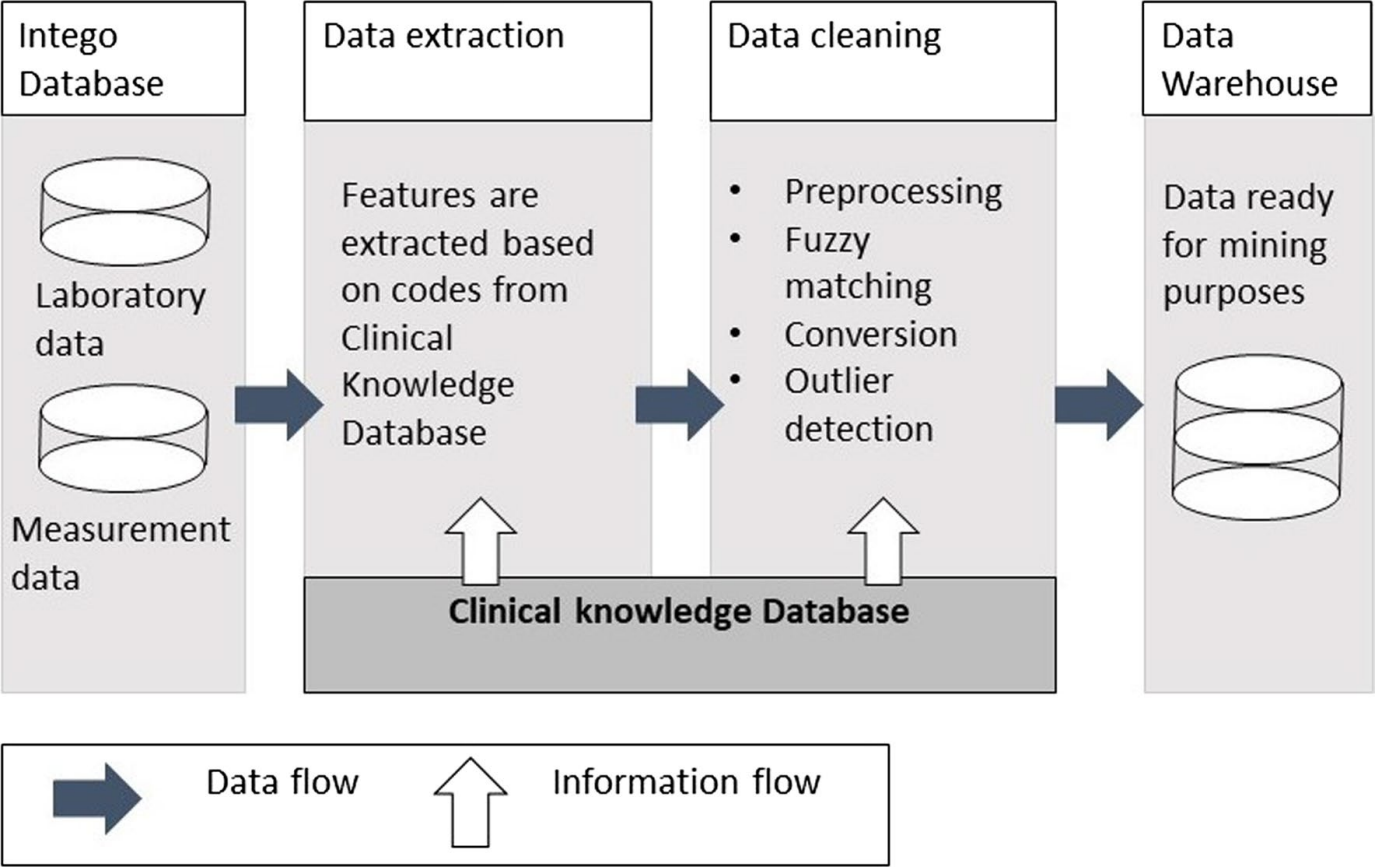
The data cleaning framework to build a data warehouse to be used for follow-up studies

With the techniques mentioned above, an automated data cleaning method was designed which could clean the data following the real clinical practice (Fig. 3).

The records of one specific variable were extracted from the whole database by using the *Lab Code* in CKD, then the preprocessing procedure was conducted to make a preliminary preparation, such as removing unexpected characters from the values, converting the values as numeric format and so on. The follow-up data cleaning procedures, i.e. fuzzy search to correct the wrongly spelled units, unit conversion, and outlier detection, were done as described in previous sections. Afterwards the cleaned variable can be stored in the data warehouse for follow-up studies.

The Clinical Knowledge Database, the codes for the automated data cleaning method, and the complete list of completeness, correctness, and plausibility can be found in Section Availability of data and materials

### Evaluation measures

There is no consensus how to evaluate data quality, especially for EHR data, but some studies gave their own definitions of dimensions of Quality of Data (QoD) for EHR data. A systematic review in 2013 summarized 5 dimensions, i.e. completeness, correctness, concordance, plausibility, and currency [2]. Completeness is the percentage of missing data, and correctness is the percentage of correct and accurate data. Concordance means comparability and consistency between data elements, for example, the prescriptions taken by the patients are in correspondence with the diagnosis. Plausibility means validity and credibility, which can be tested by checking the percentage of values falling within the biologically plausible range, i.e. the extreme values in our paper. Currency evaluates the timeliness and regency of the data. A study in 2017 summarized the quality dimensions as accuracy, completeness, consistency, credibility, and timeliness [3], while a study in 2019 proposed a model to assess primary care EHR data, covering comparability, completeness, correctness, and currency [4].

The concordance/comparability/consistency and currency/timeliness of data is not affected by our data cleaning method, so they are not evaluated in the paper. In order to evaluate other dimensions, we calculated the percentage of missing values for completeness, and the percentage of values within the normal range for correctness. Any observations outside the extreme ranges have been detected as outliers and replaced with NA, therefore, all the values are biologically plausible and plausibility is always guaranteed.

## Results

**Fig. 4:**
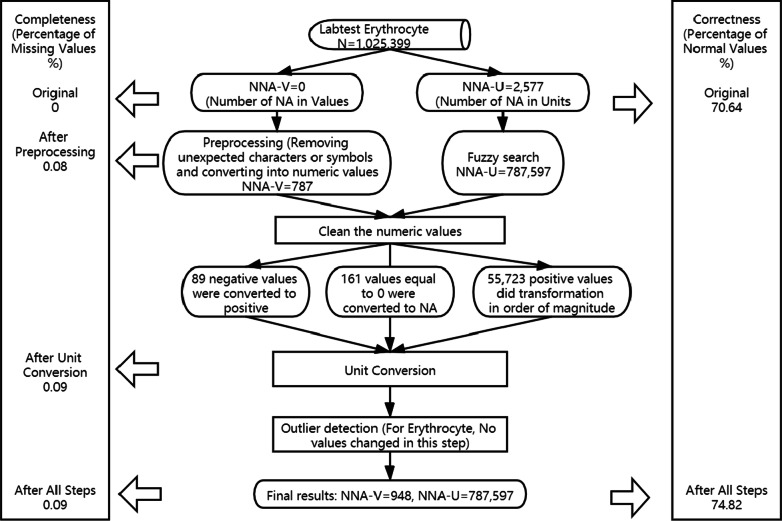
The flowchart of erythrocyte to give an example of the changes in each step

The flowchart of the whole cleaning method is shown in Fig. 4, using Erythrocyte as an example to give an idea how the variables were affected in each step. Table 4 shows the percentage of missing values and the percentage of values within the normal range before and after applying the automated data cleaning method. Only the top 10 variables with the largest volume are shown in the table, the full table has been uploaded online (See Availability of data and materials). There were no empty cells in the column for numeric values in the original data because of database maintenance, so the initial missing rates for all variables were 0. However, there were some cells with meaningless information, e.g. “,”, “n”, “NULL”, “na”, which were detected and converted into NA in the first step, data preprocessing. The completeness was not improved by using our method, because we didn’t impute missing values to increase completeness, on the contrary, it slightly decreased because we replaced the outliers with NA. All variables were 100% complete before data cleaning. In total, 42 variables had a drop less than 1% in the percentage of missing values and 9 variables declined by 1–10%. Only 1 variable experienced large decline in completeness (13.36%).

**Table 4:** Completeness and correctness before and after data cleaning (top 10 variables with the largest volume)

Test name	Completeness: percentage of missing values (%)				Correctness: percentage of normal values (%)		Number of observations
	Original	After preprocessing	After unit change	After all steps	Original	After all steps	
Hemoglobin	0	0.02	0.02	0.03	92.68	92.71	1,061,333
Lymphocyte	0	0.03	0.03	0.04	14.23	54.10	1,060,664
Eosinophils	0	0.03	0.03	3.87	15.71	96.43	1,055,109
Monocyte	0	0.03	0.03	0.26	14.12	69.93	1,053,768
Basophil	0	0.06	0.06	0.06	17.91	73.09	1,027,615
Hematocrit	0	0.02	0.02	0.02	94.02	97.17	1,025,484
Erythrocyte	0	0.08	0.08	0.09	70.61	74.82	1,025,399
Leukocyte	0	0.10	0.10	9.70	34.04	98.21	1,013,012
MCV	0	0.03	0.03	0.24	92.53	92.80	1,003,326
MCH	0	0.03	0.03	0.24	84.16	84.42	997,867

However, a slight loss in completeness is acceptable because the correctness was largely improved. Based on the percentage of the values within normal range before and after the cleaning, the correctness of most variables were largely improved, for example, Monocyte from 14.12 to 69.93% and Leukocyte from 34.04 to 98.21%. All variables had more than 50% values within the normal range after cleaning, of which 43 variables had a percentage higher than 70%. The level of data quality in the raw data varied seriously for different variables. Some variables had good quality even before the data cleaning, such as Hemoglobin (92.68%), Hematocrit (94.02%), and MCV (92.53%). In this case, our method could not further improve the data quality by a large margin, but still lead to slight improvement, e.g. from 94.02 to 97.17% and from 92.53 to 92.8%. The correctness of 9 variables were still lower than 70% after data cleaning, of which 6 variables had an increase smaller than 5% because of the cleaning procedure.

As mentioned in Section *Evaluation Measures*, the measure that is used for plausibility is often the percentage of values within extreme range. Values outside extreme ranges were detected as outliers and replaced with NA, meaning that all the values were biologically plausible. The plausibility before data cleaning is in the online document (See Availability of data and materials).

## Discussion

We proposed an automated data cleaning method for clinical variables. One of the main contributions of this method is that it allows non-technical people to explore real-world data without needing proficient data processing techniques. Although there is abundant EHR data stored in primary care, hospitals and laboratories, the technical barrier of data cleaning leads to the fact that only a small group of people, such as statisticians and data analysts, can make full use of EHR data. Take Intego Database as an example, there are 38 million laboratory tests of about 10,000 variables. In general, it may take one proficient data scientist hours to clean one variable. Then it is almost impossible to clean all the data within a reasonable period. A massive amount of data remains untouched. However, it can also provide insights and supports for decision-making if a broader range of people, e.g. clinicians and health policy makers, can freely get access to the clean and organized data in a convenient way. Despite the high automation is at the cost of slight decline in completeness, it is still reliable to get results of general trends and associations.

Another contribution of this method is it largely improved time efficiency with a slight loss in accuracy. It can take a proficient data scientist 30–40 h to clean around 50 variables manually. The time of manual data cleaning was estimated based on the past experience when the laboratory or measurement variables were cleaned manually for previous studies [20, 21]. It took 5.2 min by using the automated data cleaning method. It will be more convincing if a group of data scientists can clean the same variables manually and compare the average time with the automated method. However, it was not possible to conduct such experiment in this study because of lack of resources.

As mentioned in Background, there are some data preprocessing software or tools that can deal with commonly observed data issues. For example, the R package dataQualityR can perform variable-level data quality checks and generates summary report [22]. But these software and tools share one common characteristic, they detect abnormal values based on the data distribution and clean the data by using some summary statistics. However, with the real-world data, when there are a considerable amount of original values as abnormal values, the data distribution already departs from the distribution of the true values. Moreover, there are clinical considerations for clinical variables when cleaning data, which is from expert knowledge and is not included in data itself. Thus, most of the data preprocessing tools cannot perform well for clinical variables automatically. Manual investigation is needed in each step to make decisions based on the data reports. Our automated data cleaning method have better clinical relevance by constructing the Clinical Knowledge Database (CKD). We cleaned the numeric values not based on the data distribution, but the objective clinical knowledge, such as normal range and extreme range of clinical variables. The use of CKD also made it possible to do unit conversion on the variable level following the clinical practice. For instance, the conversion rate between mmol/L and mg/dL for glucose is 18 while it is 11.312 for creatinine.

In fact, the current study is the initial exploration of using CKD to achieve automated data cleaning for EHR data. Therefore only the most basic cleaning procedures were included in this paper. It is possible to further expand the CKD in the future by adding more clinical knowledge. For instance, the statistical measures for each variable can be added in case of data aggregation. Mean, median, min, max, etc., different measures can be used for different variables based on specific clinical considerations. It is possible to go one step further, adding more complicated “IF–THEN” rules into CKD, to help improve consistency. For example, absolute band cell count and relative band cell count are similar concepts, the difference is that one is absolute count number and the other is the percentage. If the value of relative count (a percentage) is mistakenly entered as an absolute count, it will be detected as an outlier and replaced with NA. By adding one consistency rule in CKD, the algorithm can automatically check whether this is a percentage number with unit measurement “%”, meaning that it has been assigned a wrong variable code (See variable code in Table 2). These examples reveal the potential of CKD to help achieve better clinical relevance and consistency in the future studies. The CKD acts as an expert or dictionary and the automated data cleaning method proposed in this paper guarantees that the information in CKD is made the biggest use of without losing high level of automation.

Apart from the use of CKD, we applied the fuzzy search method to automatically clean the measurement units, which, to our knowledge, is novel for cleaning medical text data. This method has not been used in previous studies with a focus on data cleaning of EHR data. Our results have shown that it could perform well for medical text data and has a great potential to be used for unstructured data cleaning in the future. Another methodological contribution is the outlier detection method. An outlier detection method was proposed in 2005 [19], which diagnosed all the data points based on the true nature of the data, using hard and soft cut-off values. We adjusted this method to make it suitable for big data, and successfully incorporated it with CKD, which helped to achieve an automated process for medical data of different types.

Based on the literature review in Background, although there were some studies using data assessment frameworks to help identify potential data issues in the original data, hence help clean the data, the level of automation was relatively low in such studies. The assessment frameworks could systematically examine the data quality, but the decision of data cleaning still needs to be done by researchers. Manual investigation cannot be avoided in the whole process. To our knowledge, two studies [14, 15] proposed an automated data cleaning method or pipeline, but both studies have inherent limitations. Phan et al. [14] only included height and weight variables, while Tang et al. focused on data preprocessing tasks such as data extraction and data harmonization, rather than data cleaning. Our method achieved to efficiently clean different types of clinical variables, with clinical relevance taken into consideration. One limitation of this method is that data longitudinality and patient variability are not considered, making it unable to check and improve data consistency. By considering the longitudinal data from the same patients, the correctness of some variables, which have large gaps between normal range and extreme range such as total cholesterol, may be further improved. However, the current method does not have auto-correction based on the data distribution, because when the correction is done automatically following some kind of distribution without the actual investigation of data, it can be very risky considering the automated method is applied for all kinds of clinical variables. Imputation bias may be introduced because of the auto-correction. In the current method, all the changes in the values are done based on the “clinical information”, which are the objective facts independent from the database. So, the cleaning procedure will not be affected by the errors in the original values, meaning that this is the basic procedure that anyone will do in the same way. Therefore, the auto-correction based on longitudinal information is not considered in the current method to guarantee validity and credibility.

To overcome the limitation on the basis of the current method, it is reliable to add longitudinal information if it is done following some pre-defined rules instead of data distribution. As mentioned above, it is possible to add more complicated consistency rules in CKD to help improve the performance of the method, similarly, the rules about longitudinal characteristics of each variable can also be added in CKD. Here is one example to demonstrate how CKD can help improve the longitudinal outlier detection in the future studies. The normal level of cholesterol is below 200 mg/dL and the extreme maximum value is 1000 mg/dL. A patient may have a series of records, 189, 174, 700, showing that the patient had healthy records at the beginning but probably got health issue when the last record occurred. There is a risk that the record of 700 is identified as “outlier” if the auto-corrected outlier detection is conducted based on longitudinal data. In this case, we exempt such values using the CKD information as they are within the extreme values defined in CKD. Another example is the possibility of adding longitudinal rules in CKD. If a patient has a record of 702, 890, 970, 1030, the last record “1030” should be replaced with NA as it is out of the extreme range. However, it is likely that this is a patient with severe symptoms. To avoid this type of issue, a longitudinal rule may be added for this specific variable, such as “IF the former values are valid and the follow-up extreme value is closely to the former values within a certain distance THEN do not replace the extreme value with NA”. The choice of “certain distance” may be variable-specific information. In summary, defining rules to detect abnormal values in the longitudinal data requires extensive discussion with data and clinical experts, which is expected to be done in future studies.

## Conclusions

While some studies proposed a general framework to clean and assess EHR or other formats of medical data, manual investigation was still needed in the whole process, very limited research studied an automated data cleaning method or tool for EHR data. Moreover, a general automated data cleaning method or tool that is able to clean all kinds of clinical variables is lacking. Furthermore, the automated data cleaning tools in other domains are not capable to accurately clean clinical data with variable-specific information taken into consideration.

This paper proposed a method which could clean clinical data automatically and is capable to work on large-scale data. Primary care EHR data was used to test the method. Results showed that with a slight loss in completeness, the credibility, correctness, and plausibility of the data were largely improved after the data cleaning. The method can be used and flexibly adjusted to clean EHR data in other studies in the future.

## Data Availability

The data that support the findings of this study are available from Intego but restrictions apply to the availability of these data, which were used under license for the current study, and so are not publicly available. The Contact person is Gijs Van Pottelbergh.

## Abbreviations

CKD: Clinical Knowledge Database
EHR: Electronic Health Records
GP: General Practitioner
QoD: Quality of Data
